# The Salutary Blood Pressure of a Solitary Kidney

**DOI:** 10.1093/ajh/hpaa004

**Published:** 2020-01-07

**Authors:** Dag Olav Dahle, Geir Mjoen

**Affiliations:** Department of Transplant Medicine, Oslo University Hospital, Oslo, Norway

The arterial media in chronic kidney disease is characterized by degradation of elastic fibers, increased collagen content and calcification, resulting in stiffening of the vessel wall.^1^ In the aorta, such wall stiffness is accompanied by increased pulse pressure due to reduced “cushioning” effect of ventricular ejection pressure. There is an associated increase in cardiac afterload, contributing to left ventricular hypertrophy and subendocardial ischemia, with increased risk of heart failure and ventricular arrhythmias. A higher pulse pressure is also associated with damage to the microcirculation in the brain and kidneys.

Aortic stiffness is a normal aging phenomenon, which is accelerated by cardiovascular risk factors such as hypertension, diabetes, and chronic kidney disease. It predicts cardiovascular disease and mortality, both in the general population and in patients with hypertension, diabetes, kidney disease, and in kidney transplant recipients.^2,3^ However, it is not known if these associations are truly causal, if aortic stiffness is reversible and by what means, and if such interventions are beneficial. Also, the mechanisms contributing to aortic stiffness are complex and the role of chronic kidney disease not fully understood.^4^

Several studies indicate that successful kidney transplantation reduces aortic stiffness,^5^ which may contribute to the lower cardiovascular risk compared with staying wait-listed in dialysis. This finding may, however, depend on improved control of volume and blood pressure after transplantation. In parallel, all antihypertensive medications reduce aortic stiffness passively by unloading the stiffer components of the arterial wall.^6^ Interestingly, donor nephrectomy was recently associated with increased aortic stiffness,^7^ indicating loss of kidney function *per se* may impact the cardiovascular system.

In this issue of American Journal of Hypertension, Buus *et al.*^8^ provide data on aortic stiffness and peripheral vascular resistance before and one year after both renal transplantation and kidney donation. Aortic stiffness was measured by pulse wave velocity (PWV), which for practical reasons is considered the reference method.^2^ In brief, each left ventricular contraction expels both a flow and a pressure wave, the latter traveling faster in stiffer aortas. Noninvasive applanation tonometry at the carotid and femoral arteries was used to calculate aortic PWV. The authors also report on other parameters calculated from the pulse waveforms: augmentation index, which is a measure of reflected pulse waves, but only partly dependent on arterial stiffness; and, excess pressure (*P*excess), a novel measure thought to represent surplus work by the left ventricle. Last, they report on forearm vascular resistance using venous occlusion plethysmography, a measure of structural changes in smaller muscular arteries.

The findings among recipients were an improvement in blood pressure and concomitant lowered augmentation index, though no significant change in PWV, *P*excess, or forearm vascular resistance. The most notable vascular change was seen in the recipients: one year after transplantation the use of antihypertensive medication was substantially reduced, and on top of this, there was a reduction in mean 24-h blood pressure of 5 mm Hg. The lack of concomitant improvement in PWV is surprising, though probably explainable by a higher variance in measurements in the recipients than in the donors. The combination of less volume overload and changes in antihypertensive medications may contribute to this. Since the transplant population is heterogeneous, with comorbidities being common, it is possible that other conditions associated with vascular stiffness are still present after transplantation and could affect vascular resistance. Secondly, if transplantation occurs after many years of chronic kidney disease and dialysis, it is likely that there is a large degree of irreversible vascular calcification. Thirdly, if there is publication bias in the existing literature, the total impression of published cohorts may be an overestimate of the true effects. Consequently, the effects on different vascular resistance measures in this population may be smaller than expected from published cohorts. This may have caused the power analyses to be based on too optimistic assumptions, rendering the final analyses underpowered to detect a true difference, due to inclusion of too few study subjects.

In kidney donors, there were no changes in blood pressure, augmentation index, *P*_excess_, or forearm resistance, but there was a small increase in PWV. From a clinical perspective, the relatively short (1-year) time span of observation warrants caution from drawing firm conclusions. We know from previous studies on kidney donors that blood pressure does not tend to rise until after several years of observation time.^9–11^ In addition to this, a sample size of 51 donors may have been too small to detect smaller increases in blood pressure. As in the study by Moody *et al.* the authors found increases in vascular resistance measurements without concurrent changes in blood pressure.^7^ A cross-sectional controlled study from 2006 by Bahous *et al.* also found increased aortic stiffness after kidney donation compared to healthy controls,^12^ although concurrent differences in blood pressure were not evaluated.

The clinical utility of aortic stiffness measurements is impeded by the need for dedicated expensive instruments and the time required per analysis (about 20 min), which restricts this method to the research setting. Also, in lieu of therapeutic options, these measurements are largely prognostic factors which are not to be acted upon. This is unlike blood pressure measurements. Although improved after transplantation, hypertension is common in kidney recipients. Blood pressure increases in kidney donors long term, and becomes more common with time since donation.^9^ For patients with end-stage kidney failure and their clinicians, the attention should be on optimizing antihypertensive treatment, ensuring timely access to transplantation, and providing life-long follow-up of both donors and recipients.

## DISCLOSURE

The authors declared no conflict of interest.
